# The risk of newly diagnosed cancer in patients with rheumatoid arthritis by TNF inhibitor use: a nationwide cohort study

**DOI:** 10.1186/s13075-022-02868-w

**Published:** 2022-08-09

**Authors:** Boyoon Choi, Hyun Jin Park, Yun-Kyoung Song, Yoon-Jeong Oh, In-Wha Kim, Jung Mi Oh

**Affiliations:** 1 Department of Pharmacy, College of Pharmacy and Institute of Pharmaceutical Sciences, CHA University, Pocheon-si, Gyeonggi Republic of Korea; 2 College of Pharmacy and Research Institute of Pharmaceutical Sciences, Seoul National University, 1 Gwanak-ro, Gwanak-gu, Seoul, 08826 Republic of Korea; 3 College of Pharmacy, Daegu Catholic University, Gyeongsan-si, Gyeongbuk Republic of Korea; 4 Division of Rheumatology, Department of Internal Medicine, Kangwon National University School of Medicine, Chuncheon-si, Kangwon Republic of Korea

**Keywords:** Arthritis, Rheumatoid, Biological products, Cohort studies, Drug-related side effects and adverse reactions, Neoplasms, Tumor necrosis factor inhibitors

## Abstract

**Background:**

Tumor necrosis factor (TNF) inhibitors use in patients with rheumatoid arthritis (RA) has raised safety concerns about cancer risk, but study results remain controversial. This largest nationwide study to date compared cancer risk in TNF inhibitor users to non-biologic disease-modifying anti-rheumatic drug (nbDMARD) users in Korean patients with RA.

**Methods:**

Data on all the eligible patients diagnosed with RA between 2005 and 2016 were retrieved from the Korean National Health Information Database. The one-to-one matched patients consisted of the matched cohort. The risks for developing all-type and site-specific cancers were estimated using incidence and incidence rate (IR) per 1000 person-years. Adjusted hazard ratio (HR) and 95% confidence interval (CI) were estimated using a Cox regression model.

**Results:**

Of the 22,851 patients in the before matching cohort, 4592 patients were included in the matched cohort. Treatment with TNF inhibitors was consistently associated with a lower risk of cancer than in the nbDMARD cohort (IR per 1000 person-years, 6.5 vs. 15.6; adjusted HR, 0.379; 95% CI, 0.255–0.563). The adjusted HR (95% CI) was significantly lower in the TNF inhibitor cohort than the nbDMARD cohort for gastrointestinal cancer (0.432; 0.235–0.797), breast cancer (0.146; 0.045–0.474), and genitourinary cancer (0.220; 0.059–0.820).

**Conclusions:**

The use of TNF inhibitors was not associated with an increased risk of cancer development, and rather associated with a lower cancer incidence in Korean patients with RA. Cautious interpretation is needed not to oversimplify the study results as cancer-protective effects of TNF inhibitors. A further study linking claims and clinical data is needed to confirm our results.

**Supplementary Information:**

The online version contains supplementary material available at 10.1186/s13075-022-02868-w.

## Background

Rheumatoid arthritis (RA) is a systemic autoimmune disease that leads to major comorbidities and mortality [1]. Because of the autoimmune pathogenesis of RA and the common etiology for RA and malignancy, RA has been suggested to increase the risk of cancer [2]. Elevated RA disease activity and complications have also been associated with an increased risk of cancer [3, 4]. Furthermore, the conventional treatment for RA, non-biologic disease-modifying anti-rheumatic drugs (nbDMARDs) such as methotrexate, was reported to accelerate cancer development by altering normal immunosurveillance [5].

Recently, biologic DMARDs, including tumor necrosis factor (TNF) inhibitors such as adalimumab, etanercept, infliximab, and golimumab, have been developed and enabled more effective disease control [6]. However, the introduction of TNF inhibitors has raised safety concerns about the risk of cancer. Although TNF-α is a proinflammatory cytokine involved in chronic inflammation in RA and the development and progression of cancer [7–9], it also plays an essential role in combating infection and killing tumor cells through natural killer cells and CD8 lymphocytes [10–12]. Therefore, treatment with TNF inhibitors could impair immunity and thereby increase the potential risk of infection and cancer.

Under this background, the results of previous studies on the risk of cancer in patients with RA treated with TNF inhibitors are controversial [1, 13–18]. Bongartz et al. reported a significantly higher incidence of cancer, but Wu et al. reported decreased cancer risk with TNF inhibitor treatment [1, 15]. For site-specific cancer, Raaschou et al. and Hellgren et al. reported nearly double the risk for squamous cell cancer and lymphoma [19, 20], while other studies found no association between those types of cancer and treatment with TNF inhibitors [21, 22].

This nationwide cohort study assessed the risk of all-type and site-specific cancers in Korean patients with RA treated with TNF inhibitors and compared them to those treated with nbDMARDs, using a national administrative database.

## Methods

In this study, we used data from the National Health Insurance Service-National Health Information Database (NHIS-NHID) [23], which is a longitudinal database containing the health care records and claims data of approximately 50 million national insurance subscribers covering over 96.3% of the population in South Korea [24]. This study was approved by the institutional review board of the Seoul National University Hospital (No. 1710-112-897).

### Study population

**Fig. 1:**
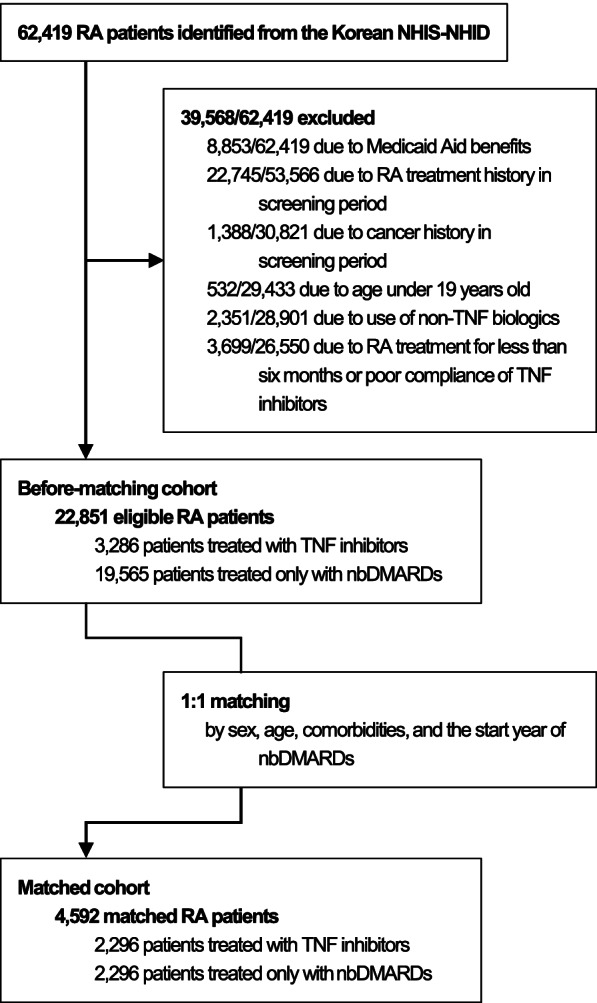
Flow diagram of study subject selection

We used a previously developed and validated algorithm for the NHIS-NHID to retrieve data from 2002 to 2016 on all patients with all M05* RA diagnostic codes of the International Classification of Diseases (ICD)-10, and a prescription of biologic DMARDs (TNF inhibitors and non-TNF biologics, including abatacept, rituximab, tocilizumab, and tofacitinib) or nbDMARDs (auranofin, azathioprine, bucillamine, cyclophosphamide, cyclosporine, D-penicillamine, hydroxychloroquine, leflunomide, methotrexate, minocycline, mizoribine, sulfasalazine, tacrolimus, and mycophenolate mofetil). The algorithm showed a high sensitivity, positive predictive value, and accuracy of 94.5, 92.4, and 90.3% for the fulfillment of 4 or more of the 1987 ACR classification criteria for RA [25]. We excluded patients receiving Medical Aid benefits (approximately 3% of the Korean population) [26] due to many missing data, with a history of RA or cancer during at least 3 years before the index date, aged under 19 years, and those using non-TNF biologics. Patients prescribed TNF inhibitors or nbDMARDs for less than 6 months and those with poor TNF inhibitors compliance, defined as the proportion of days covered (PDC) under 0.8, were excluded as well. The eligible patients constituted the before-matching cohort, representing the entire study population. The matched cohort was created by matching 1:1 TNF inhibitor users with nbDMARD users to achieve a more controlled analysis (Fig. 1). The groups were matched for age, sex, comorbidities, the Charlson comorbidity index score, and the start year of nbDMARD treatment.

Under the NHIS program, TNF inhibitor prescription is allowed only to those patients fulfilling the RA diagnostic criteria of the American College of Rheumatology (ACR) and the European League Against Rheumatism (EULAR) [27], with over 5.1 points on the disease activity score 28-joint assessment (DAS28), or 3.2–5.1 points and articular damage on radiographs, who failed to respond to at least 6 months of treatment with two or more nbDMARDs. According to this reimbursement policy and the exclusion criteria, the TNF inhibitor cohort included patients treated with TNF inhibitors for at least 6 months and nbDMARDs for at least 6 months before that. Subjects in the nbDMARDs cohort used only nbDMARDs for at least 6 months without using any biologic DMARDs.

### Follow-up

**Fig. 2:**
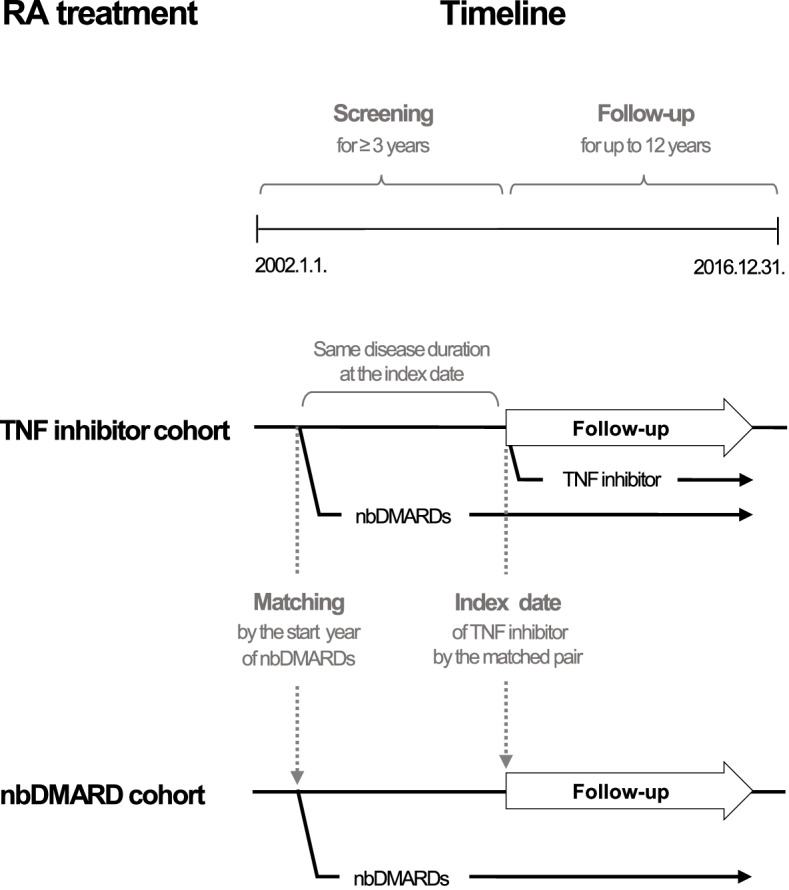
Screening and follow-up time points in each cohort

The patients were followed up from the index date to 31 December 2016 or the event date, whichever came first. Any loss to follow-up was censored. The index date of TNF inhibitor users was defined as the first date of TNF inhibitors prescription. The nbDMARD users were followed up from the index date of their matched TNF inhibitor users. The nbDMARD users in the before-matching cohort were followed up from the first date of nbDMARDs prescription (Fig. 2).

The outcome of interest was a diagnosis of cancer during follow-up. Cancer event was determined as admission to hospital with at least one of the cancer diagnostic codes and treatment codes for chemotherapy, radiotherapy, or surgery [28]. The primary outcome was all-type of cancer (ICD-10 codes C11*–C97*), and the secondary outcome was site-specific cancers (ICD-10 codes C11*–C14* for oropharyngeal, C15*–C26* for gastrointestinal, C15*–C16* and C170 for the upper gastrointestinal tract, C18*–C20* for colorectal, C22* for liver, C23*–C25* for biliary, C3* for respiratory, C40*–C41* for bone and soft tissue, C43*–C44* for skin [C43* for melanoma, C44* for non-melanoma], C50* for breast, C51*–C58* for gynecologic, C60*–C68* for genitourinary, C70*–C72* for the central nervous system, C73*–C75* for endocrine, C81*–C86*, C88*, and C90*–C96* for hematologic, and C81*–C85* and C96* for lymphoma). Incident cases within 12 months following the index date were censored.

### Confounding control

Adjusted confounding variables included age; sex; comorbidities including hypertension, diabetes, dyslipidemia, chronic liver disease (CLD), cardiovascular disease (CVD), chronic obstructive pulmonary disease (COPD), and peptic ulcer disease (PUD); the Charlson comorbidity index score [29]; disease duration; PDC by nbDMARDs, nonsteroidal anti-inflammatory drugs (NSAIDs), and oral corticosteroids; and income.

Among comorbidities, hypertension, diabetes, and dyslipidemia were identified based on ICD-10 codes (I10*–I13* and I15* for hypertension, E78* for dyslipidemia, and E10*-E14* for diabetes) plus relevant drug prescription [26]. CLD was ascertained as ICD-10 codes of viral hepatitis (B15*-B19*), alcoholic liver disease (K70*), or other CLD and cirrhosis (K73* or K74*) [30]. CVD was defined based on ICD-10 codes of ischemic heart disease (I20*-I25*), heart failure (I50*), ischemic stroke (I63*, I65* or I66*), transient ischemic attack (G45*), atherosclerosis (I70*), or aortic aneurysm (I71*) [31]. COPD was determined as age of 40 years or more, ICD-10 codes (J43* or J44*), and use of methylxanthines or inhalers for COPD [32]. Lastly, PUD was identified based on ICD-10 codes (K25*-K27*) and use of proton pump inhibitors [33]. Comorbidities and the Charlson comorbidity index score [29] were determined within 1 year of the index date. Disease duration was defined as the time from the first use of nbDMARDs to the index date. Drug treatments were recorded from the index date to the end of follow-up. The PDC was calculated as the number of days covered by prescription divided by the number of follow-up days for each patient. Patients in the top 30% and the bottom 30% income bracket at the index date were classified as high-income and low-income, respectively, while the other patients were classified as middle-income. All other variables were determined as of the index date.

### Statistical analysis

Descriptive statistics were used to summarize baseline characteristics and estimate the incidences and incidence rates per 1000 person-years for cancer. Adjusted hazard ratios (HRs) and 95% confidence intervals (CIs) were estimated by multivariable analyses using a Cox regression model (or a conditional Cox regression model for the matched cohort). The significance level was set to 0.05. Subgroup analyses were performed by age, sex, disease duration, type and duration of TNF inhibitor and nbDMARD used, and time to event. We conducted all the analyses using SAS 9.4 software (SAS Institute Inc., Cary, NC, USA).

### Sensitivity analysis and method validation

A lag time of 12 months was assumed in primary analysis for cancer development. We performed a sensitivity analysis by changing this lag time to 0, 6, 24, 36, and 60 months to assess the robustness of our findings.

We estimated the risk of tuberculosis development as a positive control outcome for method validation since it is well established that the risk of tuberculosis increases with TNF inhibitor use in patients with RA [34]. Subjects with a history of tuberculosis before the index date were excluded from the analysis of tuberculosis risk. The positive control outcome was defined based on tuberculosis ICD-10 diagnostic codes A15*–A19* and U84* and prescriptions of at least three of the anti-tuberculosis drugs following a previously developed algorithm [35]. A Cox regression model (or conditional Cox regression model for the matched cohort) was used to estimate adjusted HRs and 95% CIs after adjusting for age, sex, comorbidities (diabetes, chronic liver disease, and chronic obstructive pulmonary disease), the Charlson comorbidity index score, and the start year of nbDMARD use.

## Results

### Baseline characteristics of the study cohort

A total of 62,419 patients with RA were identified in the Korean NHIS-NHID. Of these, we excluded 8853/62,419 Medicaid Aid beneficiaries, 22,745/53,566 with RA treatment history, 1388/30,821 with cancer history during the screening period, 532/29,433 younger than 19 years, 2351/28,901 treated with non-TNF biologics, and then 3699/26,550 treated with TNF inhibitors or nbDMARDs for less than 6 months or with poor compliance with TNF inhibitor use. The remaining 22,851 patients constituted the before-matching cohort. Of these, 3286 were in the TNF inhibitor cohort and 19,565 in the nbDMARD cohort. The groups were then matched 1:1 based on the criteria described in the “Methods” section, resulting in a matched cohort of 4592 patients (Fig. 1).

The overall baseline characteristics were well balanced in the TNF inhibitor and nbDMARD cohorts, with all matching variables displaying a standardized difference value of less than 0.1 (Table 1). The mean age was 50.3 ± 13.09 years, and 78.7% were female in both cohorts after matching, similar to previous studies on the Korean RA population [13, 36].

**Table 1:** Demographic characteristics of the study cohorts

Characteristics	Before-matching cohort		Matched cohort	
	TNF inhibitor cohort (***n*** = 3286)	nbDMARD cohort (***n*** = 19,565)	TNF inhibitor cohort (***n*** = 2296)	nbDMARD cohort (***n*** = 2296)
Female gender	2298 (69.9)	14,325 (73.2)	1807 (78.7)	1807 (78.7)
Age groups	50.4 ± 14.00	57.1 ± 14.26	50.3 ± 13.09	50.3 ± 13.09
19 years	10 (0.3)	64 (0.3)	6 (0.3)	6 (0.3)
20–29 years	270 (8.2)	797 (4.1)	158 (6.9)	158 (6.9)
30–39 years	516 (15.7)	1587 (8.1)	358 (15.6)	358 (15.6)
40–49 years	656 (20.0)	3039 (15.5)	477 (20.8)	477 (20.8)
50–59 years	918 (27.9)	4890 (25.0)	706 (30.7)	706 (30.7)
60–69 years	646 (19.7)	5064 (25.9)	443 (19.3)	443 (19.3)
70–79 years	241 (7.3)	3448 (17.6)	138 (6.0)	138 (6.0)
80–89 years	28 (0.9)	668 (3.4)	10 (0.4)	10 (0.4)
90–99 years	1 (0)	8 (0)	0 (0)	0 (0)
Comorbidities^a^				
Hypertension	874 (26.6)	7100 (36.3)	474 (20.6)	474 (20.6)
Diabetes	324 (9.9)	2662 (13.6)	93 (4.1)	93 (4.1)
Dyslipidemia	521 (15.9)	3448 (17.6)	221 (9.6)	221 (9.6)
CLD	539 (16.4)	2935 (15.0)	208 (9.1)	208 (9.1)
CVD	364 (11.1)	4083 (20.9)	128 (5.6)	128 (5.6)
COPD	71 (2.2)	808 (4.1)	7 (0.3)	7 (0.3)
PUD	503 (15.3)	1982 (10.1)	0 (0)	0 (0)
Number of comorbidities^a^				
0	1649 (50.2)	8491 (43.4)	1493 (65.0)	1493 (65.0)
1	959 (29.2)	5043 (25.8)	568 (24.7)	568 (24.7)
2 or more	678 (20.6)	6031 (30.8)	235 (10.2)	235 (10.2)
Charlson comorbidity score^a^				
1	949 (28.9)	5376 (27.5)	775 (33.8)	833 (36.3)
2	936 (28.5)	4907 (25.1)	715 (31.1)	656 (28.6)
3 or more	1401 (42.6)	9282 (47.4)	806 (35.1)	807 (35.1)
Disease duration (months)^b^	33.8 ± 29.79	0 ± 0	35.6 ± 30.02	35.6 ± 30.15
TNF inhibitor treatment^c^				
Adalimumab user	1572 (47.8)	.	1089 (47.4)	.
Etanercept user	1270 (38.6)	.	882 (38.4)	.
Golimumab user	401 (12.2)	.	285 (12.4)	.
Infliximab user	673 (20.5)	.	474 (20.6)	.
Number of TNF inhibitors	1.2 ± 0.45	.	1.2 ± 0.44	.
Duration of TNF inhibitors (months)	37.6 ± 25.15	.	38.2 ± 25.23	.
PDC of TNF inhibitors^d^	0.98 ± 0.043	.	0.98 ± 0.044	.
nbDMARD treatment^c^				
Methotrexate user	2983 (90.8)	14,954 (76.4)	2136 (93.0)	1775 (77.3)
Hydroxychloroquine user	2539 (77.3)	16,343 (83.5)	1843 (80.3)	1937 (84.4)
Sulfasalazine user	2355 (71.7)	9664 (49.4)	1624 (70.7)	1180 (51.4)
Leflunomide user	1752 (53.3)	7061 (36.1)	1278 (55.7)	884 (38.5)
Number of nbDMARD	3.6 ± 1.41	3.1 ± 1.35	3.7 ± 1.37	3.3 ± 1.40
Duration of nbDMARD (months)	37.9 ± 26.60	49.8 ± 35.46	39.8 ± 26.83	35.4 ± 26.71
PDC of nbDMARD^d^	0.85 ± 0.312	0.73 ± 0.303	0.88 ± 0.288	0.82 ± 0.328
Anti-inflammatory treatment^c^				
PDC of oral corticosteroids^d^	0.73 ± 0.360	0.58 ± 0.355	0.75 ± 0.351	0.68 ± 0.383
PDC of NSAIDs^d^	0.85 ± 0.262	0.64 ± 0.340	0.86 ± 0.255	0.74 ± 0.348
Type of institution				
Tertiary hospital	3019 (91.9)	13,180 (67.4)	2114 (92.1)	1556 (67.8)
General hospital	178 (5.4)	2398 (12.3)	119 (5.2)	262 (11.4)
Community hospitals/clinics/others	89 (2.7)	3987 (20.4)	63 (2.7)	478 (20.8)
Department				
Internal medicine	3144 (95.7)	11,629 (59.4)	2193 (95.5)	1394 (60.7)
Orthopedic surgery	128 (3.9)	6280 (32.1)	93 (4.1)	708 (30.8)
Other	14 (0.4)	1656 (8.5)	10 (0.4)	194 (8.4)
Income				
High	926 (28.2)	5767 (29.5)	639 (27.8)	570 (24.8)
Intermediate	1331 (40.5)	7971 (40.7)	942 (41.0)	981 (42.7)
Low	1029 (31.3)	5827 (29.8)	715 (31.1)	745 (32.4)
Follow-up duration (years)	3.8 ± 2.3	6.0 ± 3.3	3.9 ± 2.3	3.7 ± 2.3

^a^Comorbidities and the Charlson comorbidity index scores were determined within one year of the index date

^b^Disease duration was defined as the time from the first use of nbDMARDs to the index date

^c^Drug treatments were determined from the index date to the end of follow-up

^d^PDC was calculated as the number of days covered by prescription divided by the number of follow-up days for each patient

Data are presented as mean ± standard deviation or *n* (%)

*TNF* tumor necrosis factor, *nbDMARDs* non-biologic disease-modifying anti-rheumatic drugs, *CLD* chronic liver disease, *CVD* cardiovascular disease, *COPD* chronic obstructive pulmonary disease, *PUD* peptic ulcer disease, *PDC* proportion of days covered, *NSAIDs* non-steroidal anti-inflammatory drugs

### Association between TNF inhibitor use and cancer development

The newly diagnosed cancer incidence rate per 1000 person-years in the TNF inhibitor and nbDMARD cohorts was 6.5 and 15.0, respectively, before matching and 6.5 and 15.6 after matching. The proportion of event-free patients for the before-matching and matched cohort was estimated using the Kaplan Meyer analyses (Figure S1). Multivariable analysis found TNF inhibitor use to be consistently associated with a low risk of cancer development (adjusted HR, 0.492; 95% CI, 0.351–0.688 before matching; adjusted HR, 0.379; 95% CI, 0.255–0.563 after matching; Table 2). Cardiovascular disease showed a negative association with cancer risk (adjusted HR, 0.867; 95% CI 0.761–0.987), while higher age, male sex, presence of chronic liver disease, and high PDC by nbDMARDs, corticosteroids, and NSAIDs were associated with an increased risk of cancer development. Among these, PDC by corticosteroids was also associated with a high risk of developing cancer in the matched cohort (adjusted HR, 4.418; 95% CI, 1.495–13.055; Table S1).

**Table 2:** Incidence rates and adjusted hazard ratios for cancer development

	Before-matching cohort		Matched cohort	
	TNF inhibitor cohort (12,491.9 PY)	nbDMARD cohort (117,735.9 PY)	TNF inhibitor cohort (8884.8 PY)	nbDMARD cohort (8422.2 PY)
Event number	81	1769	58	131
IR (1000 person-years)	6.5	15.0	6.5	15.6
aHR (95% CI)	0.492 (0.351, 0.688)		0.379 (0.255, 0.563)	

*TNF* tumor necrosis factor, *nbDMARD* non-biologic disease-modifying anti-rheumatic drug, *PY* person-years, *IR* incidence rate, *aHR* adjusted hazard ratio, *CI* confidence interval

### Association between TNF inhibitor use and site-specific cancer development

The multivariable Cox regression analyses on site-specific cancer in the before-matching cohort found adjusted HRs (95% CIs) in the TNF inhibitor users were significantly lower than in the nbDMARD users for gastrointestinal (adjusted HR, 0.432; 95% CI, 0.235–0.797), breast (adjusted HR, 0.146; 95% CI, 0.045–0.474), and genitourinary (adjusted HR, 0.220; 95% CI, 0.059–0.820) cancers. No significant association was observed between TNF inhibitor use and site-specific cancer development in the matched cohort. The risk of skin cancer or hematologic malignancy was not associated with TNF inhibitor use in this study (Table 3).

**Table 3:** Adjusted hazard ratios for site-specific cancer development

Cancer Site	Event number				aHR (95% CI)	
	Before-matching cohort		Matched cohort		Before-matching cohort	Matched cohort
	TNF inhibitor cohort (12,491.9 PY)	nbDMARD cohort (117,735.9 PY)	TNF inhibitor cohort (8884.8 PY)	nbDMARD cohort (8422.2 PY)	(130,227.8 PY)	(17,307.0 PY)
All cancer	81	1769	58	131	0.492 (0.351, 0.688)	0.379 (0.255, 0.563)
Oropharyngeal cavity	0	14	0	1	-	-
GI tract	25	536	18	41	0.432 (0.235, 0.797)	0.670 (0.367, 1.223)
Upper GI tract	8	149	7	11	0.846 (0.296, 2.421)	-
Colon and rectum	9	163	6	14	0.385 (0.132, 1.124)	-
Liver	4	159	3	12	0.377 (0.088, 1.616)	-
Biliary tract	6	106	3	5	0.372 (0.096, 1.436)	-
Respiratory system	19	308	13	13	0.762 (0.382, 1.522)	1.371 (0.458, 4.109)
Bone and soft tissue	1	4	0	0	8.366 (0.325, 215.255)	-
Skin	2	57	1	4	0.159 (0.014, 1.858)	-
Melanoma	1	5	0	0	0.126 (0.001, 20.528)	-
Non-melanoma	1	52	1	4	0.505 (0.023, 11.019)	-
Breast	8	316	7	58	0.146 (0.045, 0.474)	0.748 (0.090, 6.221)
Gynecologic system	8	95	6	7	1.970 (0.660, 5.883)	-
Genitourinary system	6	181	3	7	0.220 (0.059, 0.820)	-
Central nervous system	0	12	0	1	-	-
Endocrine system	9	164	8	31	0.901 (0.331, 2.456)	0.518 (0.078, 3.454)
Hematologic system	1	140	0	13	0.011 (0, 1.037)	-
Lymphoma	0	58	0	6	-	-

*aHR* adjusted hazard ratio, *CI* confidence interval, *TNF* tumor necrosis factor, *nbDMARDs* non-biologic disease-modifying anti-rheumatic drugs; *PY* person-years; *GI* gastrointestinal

### Subgroup and sensitivity analyses

The subgroup analysis by age group, sex, type and duration of TNF inhibitor and nbDMARD used, disease duration, and time to event in the matched cohort revealed that the adjusted HRs for cancer consistently tended to be lower in the TNF inhibitor cohort (Table S2). Sensitivity analysis based on various lag times of cancer development in the matched cohort demonstrated that the cancer risk tended to be consistently lower in the TNF inhibitor cohort (Table S3).

### The risk of tuberculosis development by TNF inhibitor use

The risk of tuberculosis development was confirmed to be higher in TNF inhibitor users in the matched (adjusted HR: 2.816; 95% CI, 1.243–6.383), but not the unmatched cohort (adjusted HR, 0.886; 95% CI, 0.612–1.283).

## Discussion

This is the largest study to date evaluating the risk of newly diagnosed cancer following TNF inhibitor use in Korean patients with RA. The study data included all TNF inhibitor claims in the Korean NHIS-NHID since the first TNF inhibitor was introduced to the NHIS in 2002 till 2016.

The study results indicated that the risk of cancer development was significantly lower in the TNF inhibitor cohort than the nbDMARD cohort before and after matching. This finding was consistent with several previous studies using the claims data. Wu et al. [1] and Lan et al. [14] reported adjusted HRs of 0.63 (95% CI, 0.49–0.80) and 0.59 (95% CI, 0.36–0.98), respectively, in the Taiwanese population. Cho et al. [13] reported an odds ratio (OR) of 0.42 (95% CI, 0.25–0.73) in the Korean population. Only the study by Jung et al. reported an insignificant difference in the Korean population (incidence rates ratio, 0.913; *P* = 0.546) [36]. On the other hand, meta-analyses of randomized controlled trials demonstrated an increased or insignificant risk of cancer among patients receiving TNF inhibitors compared to those taking only nbDMARDs [15, 17]. Prospective cohort studies based on the German biologics register (*Rheumatoide Arthritis: Beobachtung der Biologika-Therapie* [RABBIT]) and Australian Rheumatology Association Database (ARAD) found no difference in the risk of cancer due to TNF inhibitors use [16, 18]. In another prospective cohort study based on the Swedish biologics register (ARTIS), TNF inhibitor users did not experience more breast cancer recurrences than TNF inhibitor non-users among patients with RA and a history of breast cancer [37].

These conflicting findings could be primarily due to the differences in study design. In many studies, including the study by Jung et al. [36] that had found no difference in cancer risk due to TNF inhibitor use, patients in the two cohorts were followed up from different or random time points in their disease course. For example, subjects in the nbDMARD cohort were observed from the start date of nbDMARD treatment and those in the TNF inhibitor cohort from the start date of TNF inhibitor use. In this situation, the TNF inhibitor cohort was likely to have a longer disease duration and a longer duration of nbDMARD use since clinical guidelines and reimbursement policies allow the prescription of TNF inhibitors only to those patients refractory to nbDMARDs. Such differences could subsequently lead to a relative increase in disease activity and complications in the TNF inhibitor cohort as the disease progresses over time. The point is that not only the presence of RA has been associated with an increased risk of cancer, but also RA disease activity [3], nbDMARDs use [5], and complications such as lung disease [4]. We could control this potential bias by matching the start year of nbDMARDs use and following up each subject in the nbDMARDs cohort from the start date of the TNF inhibitor use by the matched pair (Fig. 2). This also enabled us to control the possible bias from changes in the clinical environment over time. Several new TNF inhibitors were developed during the study period, and clinical experience accumulated; therefore, biologic DMARD use has increased globally [38]. Clinical guidelines were amended many times as well. Unless controlled for, these changes over time might affect patient selection and the outcomes. This study design may be supported by many successful randomized controlled trials of TNF inhibitors in which nbDMARD users were followed-up from the start of the administration of placebo not from the start of the administration of nbDMARDs, as seen in the Anti-TNF Therapy in RA with Concomitant Therapy (ATTRACT) trial, a landmark trial of infliximab [39].

The anti-inflammatory effects of TNF inhibitors have been suggested to play a role in reducing cancer risk since chronic inflammation has been implicated in the pathogenesis of cancer [40]. TNF inhibitors were found to suppress tumor progression by disrupting TNF-α-related tumor-promoting inflammatory signaling in vitro and in vivo [41–43]. Another class of anti-inflammatory drugs, NSAIDs, has also been reported to be associated with a decreased risk of cancer, especially breast, colorectal, and genitourinary cancers [44–49]. Coincidentally, the risk of these three cancer types was shown to be significantly reduced for patients treated with TNF inhibitors in the secondary endpoint analysis of this study. It might be theoretically logical to expect that drugs exerting anti-inflammatory effects such as NSAIDs and TNF inhibitors would reduce the cancer risk by controlling chronic inflammation. However, a cautious interpretation is needed because the inference that drugs with anti-inflammatory effects would also be cancer-protective is a risky oversimplification. The exact mechanism and extent of association between cancer risk and anti-rheumatic drugs remains unclear and needs to be further researched.

Lastly, the ongoing issues surrounding cancer risk and TNF inhibitors may have shaped the behavior of physicians. For example, physicians might have avoided prescribing TNF inhibitors to patients clinically judged to be at high risk for cancer, thereby affecting the risk of cancer in TNF inhibitor users.

In regard to the factors affecting cancer risk other than TNF inhibitors, increasing age, male sex, presence of chronic liver disease, and high PDC by nbDMARDs, corticosteroids, and NSAIDs were positively associated with cancer occurrence in multivariable analysis based on cohort before matching. Age, sex, and chronic liver disease were established as carcinogenetic risk factors [50]. The high PDC by anti-rheumatic drugs might indicate a high RA disease activity, which is already known as an accelerating factor of cancer development [51]. Among the anti-rheumatic drugs mentioned above, increased use of corticosteroids remained a significant risk factor for cancer in the matched cohort. This finding is consistent with the result of a previous study on the influence of corticosteroids on the risk of skin cancer in patients with RA (adjusted OR, 2.96; 95% CI, 1.67–5.22 for cumulative doses of corticosteroids greater than 10 g) [52]. Patients with cardiovascular disease in our study were less likely to develop cancer. Similarly, Wu et al. reported a negative association between ischemic heart disease and cancer in patients with RA (adjusted HR, 0.70; 95% CI, 0.54–0.92) [1]. Those authors mentioned an independent association between cancer and the use of medications for ischemic heart disease, including NSAIDs, making this link worth further investigation.

This study has several limitations. Like many other studies using claims data, variables such as family history, smoking and alcohol use, body mass index, and laboratory data, especially on disease activity, were unavailable. We adjusted for the use of anti-rheumatic drugs as surrogate markers of disease activity. However, patients with unadjusted risk variables may still less be offered TNF inhibitors, lowering the cancer risk of TNF inhibitor users. Secondly, the sample size of the matched cohort was not large enough to estimate the risk of site-specific cancer. The follow-up duration was relatively short as well (3.9 ± 2.3 years and 3.7 ± 2.3 years for TNF inhibitor users and non-users, respectively in the matched cohort) because this was an incident RA cohort. Thirdly, we could not obtain data on the use of drugs not covered by the NHIS. Therefore, our results may have been confounded by uninsured use of TNF inhibitors by private procurement or clinical trial participation. Lastly, we used an algorithm to identify cancer [28] rather than using a high-quality cancer registry data for the outcome detection. This may have induced misclassification and selection bias. Because only events with hospital admissions were ascertained as cancers according to the algorithm, skin cancers which usually do not require hospital admissions for diagnosis were most likely to be missed especially in TNF inhibitor non-users who have less needs for hospital visits. Data linkage between claims and clinical data is needed to overcome these shortcomings of our study.

On the other hand, this study has several strengths compared to previous studies. We used nationwide administrative data for the longest period the NHIS-NHID could provide. Since Korea offers a universal health insurance service, we were able to enlarge the sample size, minimize selection bias, obtain 15 years of data, and thus enhance the statistical power to detect rare cancer events using this data source. It also permitted a solid study design. We could include only RA incident cases, account for the time of TNF inhibitor use, and match the start year of nbDMARD use. Lastly, we confirmed the validity of this study by showing the increased risk of tuberculosis in the matched TNF inhibitor cohort. In case of the before matching cohort, the lack of significance is thought to be due to the relatively less rigorous tuberculosis screening in the early study period [35], when more patients were enrolled in the nbDMARD cohort than TNF inhibitor cohort. Caution is needed in interpreting the unmatched data because of the failure to confirm an increased risk of tuberculosis as well as lack of matching. On the other hand, sensitivity analysis regarding various lag times of cancer development also confirmed the robustness of the findings. The non-significance at 60 months of lag-time may be due to the small sample size of the matched cohort (Table S3).

## Conclusions

In conclusion, we have demonstrated that TNF inhibitor use was not associated with an increased risk of cancer development, and rather associated with a decreased incidence of cancer in Korean patients with RA. Cautious interpretation is needed not to oversimplify the study results as cancer-protective effects of TNF inhibitors. Further studies with simulation or linking claims and clinical data are needed to confirm our results.

## Supplementary Information

**Additional file 1: Table S1.** Multivariable Analysis for Cancer Risk. **Table S2.** Subgroup analysis for Cancer Risk in the matched cohort. **Table S3.** Sensitivity Analysis for Cancer Risk in the matched cohort.

**Additional file 2: Figure S1.** The Kaplan Meyer curves for cancer-free proportions. **A** The Kaplan Meyer curves for the before-matching cohort. **B** The Kaplan Meyer curves for the matched cohort.

## Data Availability

The computing code required to replicate the results is provided on request. We cannot provide data of the NHIS-NHID due to data user agreement but the data could be requested from the NHIS-NHID.

## Abbreviations

RA: Rheumatoid arthritis
nbDMARDs: Non-biologic disease-modifying anti-rheumatic drugs
TNF: Tumor necrosis factor
NHIS-NHID: National Health Insurance Service-National Health Information Database
ICD-10: International Classification of Diseases-10
PDC: Proportion of days covered
NSAIDs: Nonsteroidal anti-inflammatory drugs
HRs: Hazard ratios
CIs: Confidence intervals
OR: Odds ratio
