# Correction: The risk of newly diagnosed cancer in patients with rheumatoid arthritis by TNF inhibitor use: a nationwide cohort study

**DOI:** 10.1186/s13075-022-02929-0

**Published:** 2022-10-25

**Authors:** Boyoon Choi, Hyun Jin Park, Yun-Kyoung Song, Yoon-Jeong Oh, In-Wha Kim, Jung Mi Oh

**Affiliations:** 1 College of Pharmacy and Research Institute of Pharmaceutical Sciences, Seoul National University, 1 Gwanak-ro, Gwanak-gu, Seoul, 08826 Republic of Korea; 2 Department of Pharmacy, College of Pharmacy and Institute of Pharmaceutical Sciences, CHA University, Pocheon-si, Gyeonggi Republic of Korea; 3 College of Pharmacy, Daegu Catholic University, Gyeongsan-si, Gyeongbuk Republic of Korea; 4 Division of Rheumatology, Department of Internal Medicine, Kangwon National University School of Medicine, Chuncheon-si, Kangwon Republic of Korea

**Correction: Arthritis Res Ther 24, 191 (2022)**

**https://doi.org/10.1186/s13075-022-02868-w**

Following publication of the original article [1], the authors reported that author Boyoon Choi’s affiliation “*College of Pharmacy and Research Institute of Pharmaceutical Sciences, Seoul National University, 1 Gwanak-ro, Gwanak-gu, Seoul, 08826, Republic of Korea*” was inadvertently removed.

The affiliation has been updated above and the original article [1] has been corrected.
