# Supplementary material for: The risk of newly diagnosed cancer in patients with rheumatoid arthritis by TNF inhibitor use: a nationwide cohort study
Source: Arthritis Res Ther. 2022 Aug 9;24:191. doi: 10.1186/s13075-022-02868-w (PMC9364556; doi:10.1186/s13075-022-02868-w)
Supplement: Supplementary file 1 — Additional file 1: Table S1. Multivariable Analysis for Cancer Risk. Table S2. Subgroup analysis for Cancer Risk in the matched cohort. Table S3. Sensitivity Analysis for Cancer Risk in the matched cohort. [file 13075_2022_2868_MOESM1_ESM.docx]

# **Supplemental Table 1. Multivariable Analysis for Cancer Risk**

| **Variables** | **Before-matching Cohort** | | **Matched Cohort** | |
| --- | --- | --- | --- | --- |
|  | **aHR** | **95% CI** | **aHR** | **95% CI** |
| Treatment | 0.492 | 0.351, 0.688 | 0.379 | 0.255, 0.563 |
| Age | 1.126 | 1.082, 1.172 | - | - |
| Male sex | 1.565 | 1.420, 1.725 | - | - |
| Comorbidities |  |  |  |  |
| Hypertension | 0.927 | 0.833, 1.032 | - | - |
| Diabetes | 1.041 | 0.903, 1.199 | - | - |
| Dyslipidemia | 1.008 | 0.880, 1.154 | - | - |
| CVD | 0.867 | 0.761, 0.987 | - | - |
| CLD | 1.186 | 1.046, 1.345 | - | - |
| COPD | 1.058 | 0.840, 1.332 | - | - |
| PUD | 0.980 | 0.837, 1.147 | - | - |
| Charson comorbidity score | 1.030 | 0.965, 1.098 | 1.038 | 0.760, 1.417 |
| RA disease duration | 0.997 | 0.988, 1.006 | 0.945 | 0.876, 1.019 |
| RA treatments |  |  |  |  |
| Number of nbDMARDs | 0.975 | 0.938, 1.014 | 0.964 | 0.761, 1.222 |
| PDC of nbDMARDs | 1.313 | 1.049, 1.644 | 0.592 | 0.199, 1.762 |
| PDC of corticosteroids | 1.591 | 1.330, 1.902 | 4.418 | 1.495, 13.055 |
| PDC of NSAIDs | 4.047 | 3.364, 4.868 | 1.069 | 0.393, 2.908 |
| Income |  |  |  |  |
| High vs. low | 0.976 | 0.865, 1.101 | 0.844 | 0.433, 1.644 |
| Intermediate vs. low | 0.962 | 0.861, 1.074 | 0.563 | 0.301, 1.055 |

aHR, adjusted hazard ratio; CI, confidence interval; nbDMARDs, non-biologic disease-modifying anti-rheumatic drug; CVD, cardiovascular disease; CLD, chronic liver disease; COPD, chronic obstructive pulmonary disease; PUD, peptic ulcer disease; RA, rheumatoid arthritis; nbDMARDs, non-biologic disease-modifying anti-rheumatic drugs; PDC, proportion of days covered; NSAIDs, nonsteroidal anti-inflammatory drugs

# **Supplemental Table 2. Subgroup analysis for Cancer Risk in the Matched Cohort**

| **Subgroup** | **aHR** | **95% CI** |
| --- | --- | --- |
| Age group |  |  |
| Less than 50 years | 0.284 | 0.123, 0.658 |
| 50 to 59 years | 0.348 | 0.169, 0.718 |
| 60 years or more | 0.415 | 0.200, 0.860 |
| Sex |  |  |
| Female | 0.257 | 0.077, 0.855 |
| Male | 0.315 | 0.196, 0.505 |
| RA disease duration |  |  |
| Less than 12 months | 0.591 | 0.198, 1.762 |
| 12 to less than 36 months | 0.285 | 0.135, 0.604 |
| 36 months or more | 0.409 | 0.134, 1.253 |
| TNF inhibitors |  |  |
| Adalimumab user | 0.469 | 0.282, 0.780 |
| Etanercept user | 0.361 | 0.118, 1.109 |
| Infliximab user | 0.276 | 0.140, 0.542 |
| Duration of TNF inhibitor use |  |  |
| Less than 24 months | 0.410 | 0.141, 1.194 |
| 24 to less than 48 months | 0.438 | 0.216, 0.887 |
| 48 months or more | 0.281 | 0.153, 0.516 |
| nbDMARDs |  |  |
| Methotrexate user | 0.445 | 0.292, 0.677 |
| Hdroxychloroquine user | 0.435 | 0.280, 0.677 |
| Sulfasalazine user | 0.713 | 0.371, 1.370 |
| Duration of nbDMARDs use |  |  |
| Less than 18 months | 0.419 | 0.240, 0.730 |
| 18 months or more | 0.346 | 0.144, 0.827 |
| Time to event |  |  |
| Less than 48 months | 0.198 | 0.064, 0.613 |
| 48 months or more | 0.438 | 0.190, 1.008 |

aHR, adjusted hazard ratio; CI, confidence interval; RA, rheumatoid arthritis; TNF, tumor necrosis factor; nbDMARDs, non-biologic disease-modifying anti-rheumatic drugs

**Supplemental Table3. Sensitivity Analysis for Cancer Risk in the Matched Cohort**

| **Lag Time of Cancer Development** | **aHR** | **95% CI** |
| --- | --- | --- |
| 0 months | 0.294 | 0.208, 0.416 |
| 6 months | 0.312 | 0.215, 0.452 |
| 24 months | 0.329 | 0.197, 0.550 |
| 36 months | 0.403 | 0.214, 0.760 |
| 60 months | 0.525 | 0.112, 2.463 |

aHR, adjusted hazard ratio; CI, confidence interval
