# Supplementary figures and images for: The risk of newly diagnosed cancer in patients with rheumatoid arthritis by TNF inhibitor use: a nationwide cohort study
Source: Arthritis Res Ther. 2022 Aug 9;24:191. doi: 10.1186/s13075-022-02868-w (PMC9364556; doi:10.1186/s13075-022-02868-w)

**A**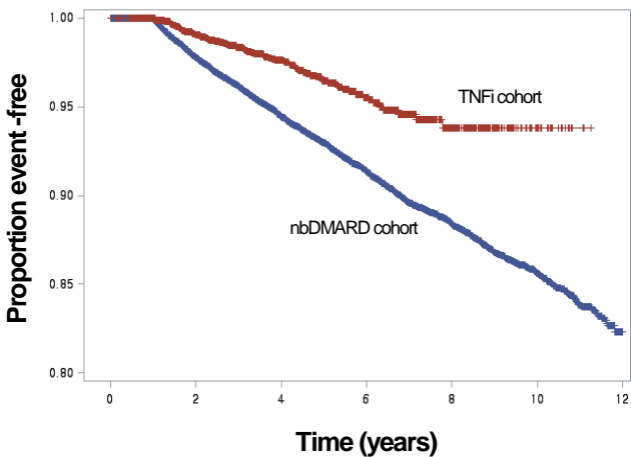**B**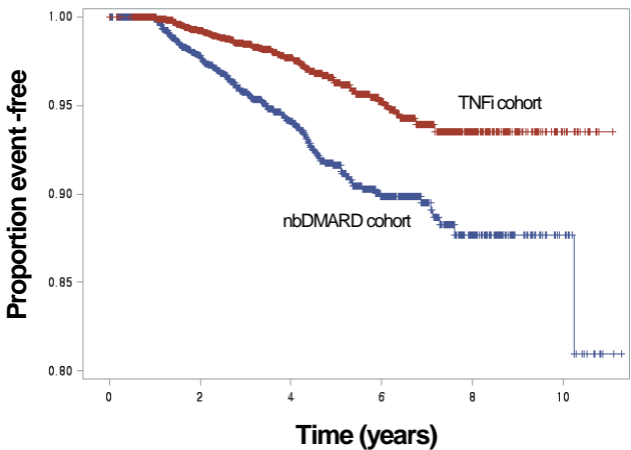

Supplement: Supplementary file 2 — Additional file 2: Figure S1. The Kaplan Meyer curves for cancer-free proportions. A The Kaplan Meyer curves for the before-matching cohort. B The Kaplan Meyer curves for the matched cohort. [file 13075_2022_2868_MOESM2_ESM.pdf]
